# Retinal changes in diabetic patients without diabetic retinopathy

**Published:** 2017

**Authors:** Alina Gabriela Dumitrescu, Sinziana Luminita Istrate, Raluca Claudia Iancu, Oana Maria Guta, Radu Ciuluvica, Liliana Voinea

**Affiliations:** * Coltea Clinical Hospital, Bucharest, Romania; ** Physiology Department I, “Carol Davila” University of Medicine and Pharmacy, Bucharest, Romania; *** Ophthalmology Department, University Emergency Hospital, Bucharest, Romania; “Carol Davila” University of Medicine and Pharmacy, Bucharest, Romania; **** “Carol Davila” University of Medicine and Pharmacy, Bucharest, Romania; ***** Anatomy Department, “Carol Davila” University of Medicine and Pharmacy, Bucharest, Romania

**Keywords:** retinal vessel caliber, optical coherence tomography, macular thickness, neurodegeneration, type 2 diabetes mellitus

## Abstract

****Purpose**.:**

The purpose of this study was to measure retinal vessel caliber and to examine early changes in macular thickness using optical coherence tomography (OCT). We evaluated to what extend vascular caliber and macular thickness differed between patients with type 2 diabetes mellitus without diabetic retinopathy compared with healthy individuals.

****Methods**.:**

26 diabetic patients without diabetic retinopathy and 26 normal participants without any retinal and optic nerve diseases underwent ophthalmic examination, fundus photography, and OCT imaging. Temporal inferior retinal vessel diameters were measured using OCT. Also, we measured macular thickness in nine ETDRS subfields using Cirrus OCT.

****Results**.:**

The mean age in the diabetic group was 61.5 years and in the control group, 55.5 years. Wider retinal arterioles and venules were found in patients with diabetes compared with healthy subjects (120 µm versus 96 µm, p<0.005 and 137 µm versus 120.5 µm, p value <0.001, respectively). In patients with type 2 diabetes mellitus, central macular thickness was significantly thinner than that of control eyes (243.5 µm versus 269.9 µm, p value <0.001).

****Conclusions**.:**

Our results support the hypothesis that the association between vascular damage and structural changes of the neuroretina is an early indicator of retinal impairment in patients with diabetes without diabetic retinopathy.

## Introduction

According to World Health Organization (WHO), more than 422 million people worldwide have diabetes mellitus (DM), and the number is increasing with an expected number of 552 million by 2030 [**1**,**2**]. Diabetic retinopathy (DR) is a frequent ocular complication and one of the leading causes of blindness in developed countries. DR is common in the first 5 years duration of type 1 diabetes and all the patients with type 2 diabetes have some form of DR after 20 years from the onset [**3**]. Thus, a protocol is needed to identify the individuals at great risk of blindness, before permanent changes in the retina occur. We need objective and reproducible tests for screening, early diagnosis and treatment evaluation of diabetic retinopathy. 

For many years, diabetic retinopathy was considered a form of vasculopathy. The osmotic stress from hyperglycemia is the pathophysiological mechanism of increased intraretinal vascular permeability and variable degrees of intraretinal capillary closure, resulting in macular edema and ischemia [**4**]. Recent studies suggest that neurodegeneration plays an important role in the pathogenesis of DR [**5**]. Histological studies of autopsy samples have revealed that the alternation of the metabolic pathways in diabetes can potentially cause neural cell degeneration in the retina [**6**,**7**]. Some of the studies reported that the neural loss might occur before any visible signs of vascular changes. Animal studies have shown that, in early stages of retinopathy, neural apoptosis, loss of ganglion cell bodies and glial reactivity can be observed [**8**,**9**]. The interaction between regulation of blood flow and neural activity is considered to be involved in the pathophysiologic mechanism of DR, and is described as neurovascular coupling [**10**,**11**]. 

Optical coherence tomography (OCT) is used for quantitative analysis of retinal architecture and provides detailed scans of retinal structure with a high resolution, retinal thickness, choroid and optic disc morphometry [**12**]. OCT images can be used to qualitatively assess pathological changes of the retina or to make quantitative measurements, in vivo [**13**,**14**]. Recently, Spectral Domain OCT has been used to measure retinal vessel diameter [**15**,**16**]. OCT has become an important tool for screening, diagnosis and treatment evaluation of diabetic retinopathy [**12**]. 

Studies have shown that vascular parameters such as venular dilation and larger arteriolar caliber are associated with DR [**17**]. Several groups have shown that total retinal thickness is decreased in patients with no or minimal DR compared with healthy controls [**18**]. OCT measures the structural composition of the neuroretina and can assess retinal neurodegeneration. 

The purpose of this study was to determine whether diabetes affects the vascular caliber and the macular thickness. We speculated that the interaction between vascular and neurogenic damage might be an early indicator of retinal impairment in patients with type 2 DM and no DR. To test this hypothesis, we measured the retinal vascular caliber and the macular thickness by OCT in diabetic patients without DR and healthy controls. 

## Material and methods

**Study population**

The data in the present prospective study were obtained from a population aged 40-90 years, examined in the Ophthalmology Clinic of the University Emergency Hospital Bucharest from August 2016 to May 2017. This study was conducted according to the recommendations of the Declaration of Helsinki and was approved by the research ethics committee of the University Emergency Hospital Bucharest. A written informed consent was obtained from all the participants. Subjects were divided into two groups: 1. With diabetes mellitus and 2. Healthy individuals. 

The inclusion criteria in the diabetes group were duration of type 2 DM for at least 5 years, but not more than 10 years, age between 40 and 90 years, without microvascular or macrovascular complications of DM and with oral or subcutaneous treatment of hyperglycemia. Healthy control subjects did not have any diagnosed ocular disease, diabetes, or other systemic disease.

Subjects with cardiovascular pathology, renal failure, diabetic neuropathy or glycosylated hemoglobin (HbA1c) > 86 mmol/ mol (10%), were excluded from the study. We also excluded eyes with diabetic retinopathy, previous laser photocoagulation, history of ocular surgery, ocular diseases that could cause degeneration (glaucoma, uveitis or retinal disease including retinal vein occlusion and age related macular degeneration) and refractive error of more than 3.00 spherical diopters and 1.00 cylindrical diopters. Patients with blurred ocular media, intraocular pressure > 21 mmHg abnormal perimetry and inadequate pupil dilatation were excluded.

All the participants passed a screening examination that included a full medical history, physical examination, blood sampling (for glycosylated hemoglobin), best corrected visual acuity measurement, slit lamp biomicroscopy, IOP measurement. After pupil dilatation with 1% tropicamide eye drops, stereoscopic retinal photographs were acquired, and macular thickness and retinal vessel caliber were measured using OCT. 

**Optical coherence tomography imaging**

All the subjects were examined with the Cirrus HD-OCT (model 400, Carl Zeiss Meditec). Cirrus is a spectral domain OCT with scan speed of 27000 A-scans/ second, scan depth of 2.00 mm (in tissue), axial resolution of 5 µm (in tissue) and transverse resolution of 15 µm (in tissue). After pupil dilation, each subject was seated in front of the OCT scanner and his head was stabilized on the chin rest. An internal fixation target was used to obtain the highest reproducibility and to avoid eye movement. Scans were performed through a dilated pupil while monitoring the video image of the central retina using the scanning system Live OCT fundus technology. 

**Structural neurodegeneration**

The retinal thickness is defined as the distance between the vitreoretinal interface and the anterior surface of the retinal pigment epithelium along each A-scan. The “fast macular thickness” OCT protocol was performed to quantify retinal structural changes. This scan protocol consists of six radial scans of 6 mm length, equally spaced at 30o and centered on the fovea. The scanned retina was divided in three areas defined according to Early Treatment Diabetic Retinopathy Study (ETDRS). The areas were: (1) the fovea, the central circle with a diameter of 1 mm; (2) the pericentral area, a donut-shaped ring centered on the fovea, that had an inner diameter of 1 mm and an outer diameter of 3 mm; and (3) the peripheral area, with an inner diameter of 3 mm and an outer diameter of 6 mm. Inner and outer zones were divided in four quadrants: superior, nasal, temporal and inferior. Macular thickness was defined as the average value of all the scans in every retinal quadrant. 

**Retinal vessel caliber measurement**

We used a 5-line raster placed 1 mm above the inferior border of the optic disc to scan the retinal vessels. After obtaining the OCT images, we chose the temporal inferior artery and vein aligned perpendicular to the scan, and manually measured the lumen diameter using the “straight” tool. 

**Fundus photography imaging**

Mydriatic Zeiss camera was used to acquire optic disc centered fundus color photographs with a stereoscopic 35-degree field, according to the Fundus Photograph Reading Center procedure [**19**]. Fundus photography excluded from the study eyes with diabetic retinopathy. 

**Statistical analysis**

EXCEL and SPSS 22.0 software was used for statistical analysis. Descriptive statistical data were described as mean and categorical data and were presented as percentages. Total retinal thickness in every quadrant was recorded for the DM and healthy groups. Also, arteriolar and venular calibers were recorded for the two groups. The Mann-Whitney U test was used to test the differences in continuous data between the study groups. The P value < 0.05 was considered statistically significant.

## Results

In total, 52 subjects were included in the study, of whom 26 (14 women and 12 men) had diabetes mellitus without diabetic retinopathy (group 1) and 26 (20 women and 6 men) healthy controls (group 2). The mean age of the subjects in the two groups was 61.5 and 55.5 years, respectively. The clinical data and demographics of the patients and controls are shown in **Table 1**. 38.46% of the patients in group 1 were treated with insulin and 61.54% had oral hypoglycemic therapy. 

**Table 1:** Clinical characteristics (median values) of subjects

	Group 1 (DM) n=26		Group 2 (control) n=26	
Age (years)	61.5		55.5	
Predominant age group (years) (%)	60-70	35%	40-50	35%
Sex (female: male)	14:12	54%:46%	20:6	77%:23%
Home environment	Urban	62%	Urban	69%

**Retinal vessel caliber analysis**

**Fig. 1:**
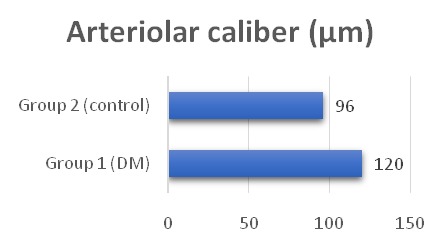
Variation of arteriolar caliber

**Fig. 2:**
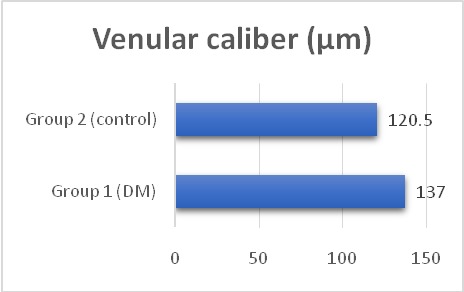
Variation of venular caliber

The average arteriolar diameter was 120 µm in the diabetic group and 96 µm in the control group (**Fig. 1**). The average venular diameter was 137 µm in group 1 and 120.5 µm in group 2 (**Fig. 2**). 

There was a statistically significant difference in arteriolar and venular caliber between the patients with diabetes and the healthy controls (U=189, p<0.005 and U=96.5, p<0.001, respectively). Both the arteriolar and venular calibers were wider in the diabetic group (**Table 2**).

**Table 2:** Mann-Whitney U test results: comparison of retinal arteries and veins in diabetic and non-diabetic patients

	Test Statistics a	
	Artery	Vein
Mann-Whitney U	189,000	96,500
Wilcoxon W	540,000	447,500
Z	-2,731	-4,428
Asymp. Sig. (2-tailed) (P value)	,005	,000

Macular thickness analysis

The mean macular thickness at the central sector in the diabetic group was significantly thinner than in the control group (U=99; p<0.01). Retinal thickness in fovea center was 243.5 µm in group 1 and 269.5 µm in group 2. Except for the center, the macular thickness in all quadrants in the diabetic patients was not statistically significantly different from the control group (**Table 3** and **4**).

**Table 3:** Mean macular thickness in all 9 ETDRS regions. * p value <0.001

	Group 1 (µm)	Group 2 (µm)
Foveal center (ETDRS central region: R1)*	243.5	269.5
*Inner circle (ETDRS pericentral region)*		
Superior (R2)	319	316
Nasal (R3)	313	317
Inferior (R4)	309	312
Temporal (R5)	308	300
*Outer circle (ETDRS peripheral region)*		
Superior (R6)	270	280
Nasal (R7)	286	292
Inferior (R8)	264.5	267.5
Temporal (R9)	254.5	264.5

## Discussions

Because diabetic retinopathy is a common complication of diabetes, an early diagnosis and proper management can reduce the incidence of vision loss. The new OCT devices are the proper tool for an easy and repeatable evaluation of the retina. 

Many of previous published studies were based on the effect of vascular changes on visual function in patients with diabetic retinopathy [**20**,**21**]. Some authors reported impaired contrast sensitivity in diabetic patients with normal visual acuity and minimal diabetic retinopathy [**22**,**23**]. Hyperglycemia can cause endothelial dysfunction due to inflammation and retinal hypoxia [**24**,**25**]. Previous studies reported that incidence and progression of DR is associated with wider retinal venular diameters [**26**,**27**]. Studies reported different results regarding the arteriolar caliber. Some authors showed that narrower retinal arterioles can predict the incidence of proliferative DR [**28**], while others found that a wider arteriolar caliber is correlated with the development of incipient DR [**29**]. 

In this study, we reported that patients with DM have a wider retinal vascular caliber compared with healthy controls, both venular and arteriolar. The arteriolar caliber increased from 96 µm in healthy subjects to 120 µm in diabetic patients. Venular diameter also increased from 120.5 µm in the control group to 137 µm in the diabetic group. The difference was statistically significant (p value < 0.005 for arteriole and < 0,001 for venule). These changes might have been caused by impaired vascular autoregulation and ischemia. Our results were similar with other studies.

**Table 4:** Mann Whitney U test results: comparison of retinal thickness in diabetic patients and non-diabetic patients

					Test Statistics a				
	R1	R2	R3	R4	R5	R6	R7	R8	R9
Mann-Whitney U	99,000	300,500	299,500	318,500	297,000	337,000	329,000	267,500	287,500
Wilcoxon W	450,000	651,500	650,500	669,500	648,000	688,000	680,000	618,500	638,500
Z	-4,376	-,687	-,705	-,358	-,752	-,018	-,165	-1,291	-,925
Asymp. Sig. (2-tailed) (P value)	,000	,492	,481	,721	,452	,985	,869	,197	,355

Because OCT evaluates the morphology of the retina, retinal neurodegeneration can be assessed clinically. Only a few clinical studies have investigated the neurogenic damage in patients with diabetes. The first study reported an increased retinal thickness of the superior nasal quadrant of the macula in patients with DR compared with patients without DR and controls [**30**]. Another study suggested that localized areas of retinal thickening can be found in initial stages of diabetic retinopathy [**31**]. One report found that fovea was significantly thinner with a longer duration of disease in a patient with no or mild DR [**32**]. Two other groups concluded that the pericentral retinal thickness was significantly decreased and reported a subnormal Rarebit Fovea Test result in patients with minimal DR [**18**,**33**]. 

In our study, we found a decreased fovea thickness in diabetic patients without diabetic retinopathy compared with healthy controls. Macular thickness in the center of the fovea was 243.5 µm in the diabetic group and 269.5 µm in the control group. The difference between groups was statistically significant (P value <0.001). The difference was not statistically significant between groups in all the other macular quadrants.

Several studies reported that retinal neuronal abnormalities are present in the early stages of diabetes [**8**,**34**-**36**]. With an increase in the duration of DM, neuronal abnormalities (retinal ganglion cells death and axonal degeneration) are the cause for reduced RNFL thickness. If neuronal abnormalities develop before the increase of vascular permeability, these changes may explain the thinner macular thickness in early stages of diabetes. Further studies are needed to determine if changes in RNFL are the cause of the reduction in macular thickness. 

## Conclusions

In the early stage of diabetes, the retinal vascular caliber and central macular thickness, measured with OCT, are altered in patients without diabetic retinopathy. Both arterioles and venules are wider in diabetic patients compared with healthy subjects. Central fovea thickness is reduced in patients with diabetes mellitus. 

We used OCT to study the human retina in vivo and non-invasively. We concluded that the retinal vessel caliber changes combined with structural retinal neurodegeneration might be an early indicator of retinal impairment in diabetes, before clinical evidence of diabetic retinopathy.

We believe that intraretinal examination with OCT might help understand the macular pathophysiology and might become an important diagnostic tool in diabetic patients with no clinical evidence of retinopathy.

**Conflict of interest statement**

The authors declare that they have no conflict of interest.
